# Different alternative splicing patterns are subject to opposite selection pressure for protein reading frame preservation

**DOI:** 10.1186/1471-2148-7-179

**Published:** 2007-09-28

**Authors:** Feng-Chi Chen, Trees-Juen Chuang

**Affiliations:** 1 Division of Biostatistics and Bioinformatics, National Health Research Institutes, Miaoli County 350, Taiwan; 2 Genomics Research Center, Academia Sinica, Taipei 11529, Taiwan

## Abstract

**Background:**

Alternative splicing (AS) has been regarded capable of altering selection pressure on protein subsequences. Particularly, the frequency of reading frame preservation (FRFP), as a measure of selection pressure, has been reported to be higher in alternatively spliced exons (ASEs) than in constitutively spliced exons (CSEs). However, recently it has been reported that different ASE types – simple and complex ASEs – may be subject to opposite selection forces. Therefore, it is necessary to re-evaluate the evolutionary effects of such splicing patterns on frame preservation.

**Results:**

Here we show that simple and complex ASEs, respectively, have higher and lower FRFPs than CSEs. Since complex ASEs may alter the ends of their flanking exons, the selection pressure on frame preservation is likely relaxed in this ASE type. Furthermore, conservation of the ASE/CSE splicing pattern increases the FRFPs of simple ASEs but decreases those of complex ASEs. Contrary to the well-recognized concept of strong selection pressure on conserved ASEs for protein reading frame preservation, our results show that conserved complex ASEs are relaxed from such pressure and the frame-disrupting effect caused by the insertion of complex ASEs can be offset by compensatory changes in their flanking exons.

**Conclusion:**

In this study, we find that simple and complex ASEs undergo opposite selection pressure for protein reading frame preservation, with CSEs in-between. Simple ASEs have much higher FRFPs than complex ones. We further find that the FRFPs of complex ASEs coupled with flanking exons are close to those of simple ASEs, indicating that neighboring exons of an ASE may evolve in a coordinated way to avoid protein dysfunction. Therefore, we suggest that evolutionary analyses of AS should take into consideration the effects of different splicing patterns and the joint effects of multiple AS events.

## Background

Alternative splicing (AS) is a topic of increasing interests because it has been suggested to be an important contributor to transcriptome/proteome complexity, gene function, and a wide variety of biological processes [1-7]. Previous studies have reported that as high as 40~80% of human genes undergo AS [8-12]. Of the observed AS events in mammals, the most common AS event is "cassette exon". It can add or remove an individual exon in a transcript [13-15]. Cassette exons are sometimes referred to as alternatively spliced exons (ASEs) [16-23]. It has been suggested that ASEs and constitutively spliced exons (CSEs, exons that are always included in the transcript) are under different selection pressures and evolve at distinct rates – the former have higher nonsynonymous (*Ka*) substitution rates but lower synonymous (*Ks*) substitution rates than the latter [16,18,19,24-26]. ASEs are regarded as under relaxed selection pressure because of their dispensability in transcripts. Also, conserved ASEs (i.e., exons are alternatively spliced in a pair of compared species) have been suggested to be constrained for preservation of the reading frame [18,22,27]. Many studies have pointed out that preservation of reading frame may indicate functional selection pressure of an AS event [18,22,27-29].

**Figure 1:**
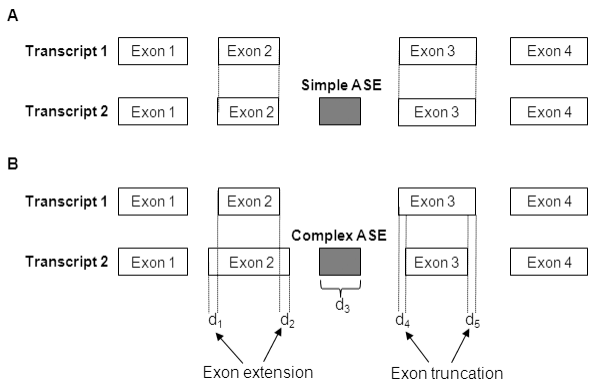
Two kinds of ASEs analyzed in the study (A) simple ASEs and (B) complex ASEs. Complex ASEs change the boundaries of one or both of their flanking exons when they are included in transcripts while simple ASEs do not. Therefore, a complex ASE looks like a simple ASE plus exon extension/truncation events. The length difference between Transcripts 1 and 2 is d_1 _+ d_2 _+ d_3 _- d_4 _- d_5_.

Recently, the Alternative Splicing Database (ASD) project at European Bioinformatics Institute (EBI) [30] further classifies cassette exons into simple and complex cassette exons ("simple ASEs" and "complex ASEs"). Complex ASEs differ from simple ones in that the former change the lengths of one or both of their flanking exons when they are included in the transcripts, whereas the latter do not (see Fig. 1). Therefore, inclusion of a complex ASE results in simultaneous changes of two or three exons. In contrast, inclusion of a simple ASE does not alter its flanking exon(s) and appear to cause fewer changes. Chen *et al*. have reported that simple ASEs have higher *Ka *and lower *Ks *than CSEs, whereas complex ASEs have evolutionary rates to the opposite of simple ASEs vs. CSEs [31]. They also found that GC contents and codon usage bias are associated with increased *Ks *values in complex ASEs but not in simple ones [31]. Such observation modified the previous view that ASEs accelerate evolution of protein subsequences. However, whether simple/complex splicing pattern is related to preservation of reading frame has not been investigated.

## Results and discussion

**Figure 2:**
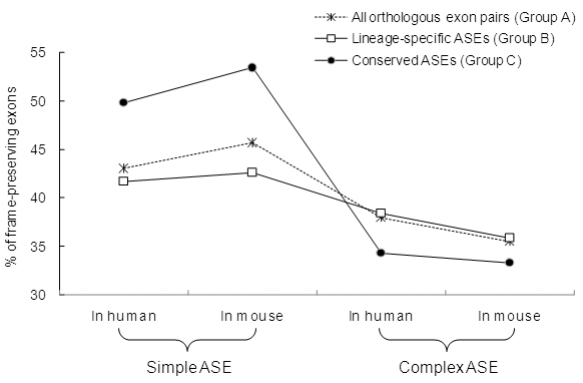
Comparisons of frame-preserving frequencies of simple ASEs and complex ASEs in ASE conservation unspecified group (Group A), lineage-specific ASE group (Group B), and conserved ASE group (Group C).

**Figure 3:**
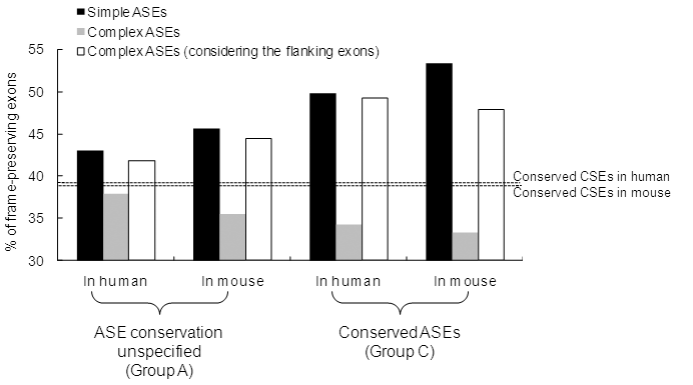
Comparisons of frame-preserving frequencies of simple ASEs, complex ASEs, and complex+ flanking exons in Groups A and C.

Since ASEs in one species may be CSEs in the other (i.e. lineage-specific ASE/CSEs), it is necessary to specify the splicing pattern of the exons studied. Therefore, we classify ASEs into three major groups according to splicing pattern conservation (see Materials and Methods). Each group is subsequently divided into four subsets (Table 1). We then compare the frequencies of reading frame preservation (designated as "FRFP", i.e., the proportions of exons of which the lengths are divisible by 3) between simple and complex ASEs (Fig. 2). For Group A, the FRFPs for human (mouse) simple and complex ASEs are 43.0% (45.7%) and 37.9% (35.5%), respectively. In comparison, the FRFPs of CSEs approximate 40% (39.7% in human and 39.5% in mouse [22]). It has been well recognized that CSEs have lower FRFPs than ASEs. However, we find that although this is true for simple ASEs (*P*-values < 0.01 in both human and mouse; all statistical tests used in this section are the Fisher's exact test), it does not seem to hold for complex ASEs. The FRFPs are higher in CSEs than in complex ASEs in both species, though the differences are not highly significant (both *P*-values > 0.01). Overall, our results indicate that simple and com1plex ASEs are under opposite selection pressure for protein reading frame preservation. Particularly, complex ASEs differ significantly in FRFP from commonly regarded ASEs, which are dominated in number by simple ASEs. We then extract conserved ASEs (Group C) from Group A. Note that "conservation" here refers to the conservation of the ASE/CSE splicing pattern between human and mouse, rather than the simple/complex pattern. We find that the FRFP of Group C simple ASEs increases to 49.8% in human and 53.4% in mouse (Fig. 2). Meanwhile, for Group C complex ASEs, the FRFPs decrease to <35% (34.3% for human; 33.3% for mouse) (Fig. 2). It is obvious that simple ASEs in Group C have higher FRFPs than in Group A, whereas the reverse is true for complex ASEs in both human and mouse. We then compare the FRFPs of simple and complex ASEs with those of CSEs. For simple ASEs, Group A has lower FRFPs than Group C, while both groups have higher FRFPs than those of CSEs (Fig. 3). However, for complex ASEs, the trend is reversed. Even if the expected FRFP of CSEs is set as 45% [32], the trends still hold well in conserved ASEs. Therefore, simple and complex ASEs seem to cause FRFP changes to the opposite ends when compared with CSEs. Note that the "CSEs" stated above are those with unspecified splicing pattern conservation. We therefore retrieve 21,669 pairs of conserved CSEs for comparison. The FRFPs of conserved CSEs are 38.4% in human and 38.3% in mouse, respectively. These figures further confirm our observations that CSEs tend to have higher FRFP than complex ASEs but lower FRFP than simple ones. Overall, our result supports Chen et al's suggestion that simple and complex ASEs cause evolutionary changes to the contrary ends with CSEs in-between [31].

**Table 1:** The retrieved human-mouse orthologous exon pairs

Human-mouse ortholog Exon types		No. of human-mouse orthologous exon pairs
**Group A: ASE conservation unspecified group**		

**Human**	**Mouse**	

Simple ASEs	All exons	1,960
Complex ASEs	All exons	311
All exons	Simple ASEs	1,299
All exons	Complex ASEs	293

**Group B: Lineage-specific ASE group**		

**Human**	**Mouse**	

Simple ASEs	CSEs	1,635
Complex ASEs	CSEs	276
CSEs	Simple ASEs	932
CSEs	Complex ASEs	254

**Group C: Conserved ASE group**		

**Human**	**Mouse**	

Simple ASEs	All ASEs	325
Complex ASEs	All ASEs	35
All ASEs	Simple ASEs	367
All ASEs	Complex ASEs	39

Note: All exons include CSEs and all ASEs. All ASEs include simple ASEs, complex ASEs, and ASE type uncertain. Lineage-specific and conserved ASE groups are subsets of the ASE conservation unspecified group.

To further probe the effects of splicing pattern conservation on frame preservation, we compare the FRFPs between conserved and lineage-specific ASEs (Groups C and B). As shown in Figure 2, for simple ASEs, conservation of ASE/CSE splicing pattern results in an increase in FRFP. In contrast, splicing pattern conservation causes the FRFP to drop in complex ASEs, such observation disobeys the previous view [18,22,27] that conserved ASEs have a higher probability to be frame-preserving than lineage-specific ones.

On the other hand, also see Table 1, we find that >70% of the ASEs (either simple or complex) have CSE counterparts in the other species, indicating that AS patterns tend not to be evolutionarily conserved in human and mouse. If only conserved ASEs are considered, the simple splicing pattern has a much higher probability of being conserved between human and mouse than the complex splicing pattern (Table 2). The result indicates that most complex ASEs are lineage-specific.

**Table 2:** Classification of conserved ASEs in terms of simple/complex splicing pattern

**Human-mouse ortholog AS types**		**No. of human-mouse orthologous exon pairs (%)**
**Human**	**Mouse**	

Simple ASEs	All ASEs	325
	Simple ASEs	244 (75.1)
	Complex ASEs	23 (7.1)
	ASE type uncertain	58 (17.8)
Complex ASEs	All ASEs	35
	Simple ASEs	25 (71.4)
	Complex ASEs	7 (20.0)
	ASE type uncertain	3 (8.6)
All ASEs	Simple ASEs	367
Simple ASEs		245 (66.7)
Complex ASEs		24 (6.5)
ASE type uncertain		98 (26.7)
All ASEs	Complex ASEs	39
Simple ASEs		23 (59.0)
Complex ASEs		7 (17.9)
ASE type uncertain		9 (23.1)

Another issue of interest is that, since a complex ASE looks like a simple event plus one (or two) exon extension/truncation event(s) (see Fig. 1B), the FRFPs of complex ASEs may in fact reflect the effects of exon extension/truncation events. However, as shown in Table 3, we find that the FRFPs in the lineage-specific exon extension/truncation events are around 50%, whereas in conserved events, the FRFPs significantly increase to over 60% (both *P*-values < 0.001; Table 3). Such an increase in FRFP towards conserved ASEs is similar to what is observed in simple ASEs. Therefore, exon extension/truncation events and complex ASEs may be under different selection pressures for reading frame preservation. We speculate that a complex splicing event is rather an integrated "module" that requires synchronized changes in neighboring exons, than merely a simple ASE accidentally coupled with exon extension/truncation events. To find support for this hypothesis, we further analyze whether the length changes caused by complex ASEs and their flanking exons can offset the frame-shifting effects of each other and retain the upstream reading frame. We find that the FRFPs of complex ASEs coupled with flanking exons (complex+flanking exons) are close to those of simple ASEs (Fig. 3). In Group C, the FRFPs of complex+flanking exons (49.2% in human and 47.8% in mouse) are significantly higher than those of conserved CSEs (dashed lines in Fig. 3; both *P*-values < 0.01). Therefore, the selection pressure for frame preservation may apply to transcripts as a whole, but not to complex ASEs *per se*. Furthermore, our results imply that in an alternatively spliced transcript, neighboring exons of an ASE may evolve in a coordinated way to avoid protein dysfunction.

**Table 3:** The frame-preserving frequencies of exon extension/truncation AS events

	No. of events	No. of events with reading frame preservation (%)
**Human**		

AS in human and CS in mouse	1077	552 (51.25)
AS in both human and mouse	294	185 (62.93)
*P*-value < 0.001		

**Mouse**		

AS in mouse and CS in human	871	416 (47.76)
AS in both mouse and human	294	194 (65.99)
*P*-value < 0.00001		

Note that "reading frame preservation" here means that the changes in exon length caused by such events are multiples of 3.

## Conclusion

In sum, one surprising finding of this study is that the FRFP of complex ASEs is lower than that of CSEs. Our result suggests that the frame-shifting effects of complex ASEs are rescued by the compensatory changes in the flanking exons, thus leaving the downstream protein reading frames unaltered. Therefore, complex ASEs appear to be more relaxed from selection pressure than simple ones in terms of reading frame preservation. One possible reason is that most observed ASEs (>80%) are simple ASEs (see Table 1) and the previously analyzed results are likely dominated by the effects of these exons. If we divide ASEs into simple and complex ASEs, the opposite evolutionary effects between them are observed. Previously, we have reported that complex ASEs are under stronger selection pressure against amino acid changes than simple ones [31]. In addition, we find that exons that participate in both simple and complex AS events have intermediate FRFPs, which fall between those of simple and complex ASEs (data not shown). In sum, our results reveal that, simple and complex ASEs have quite distinct evolutionary features. It appears that both simple and complex AS patterns have functional importance in view of the two different forms of selection pressure (protein sequence conservation and reading frame preservation) for which they are constrained. Although the biology of complex ASEs has rarely been documented, it is likely that this ASE type has resulted from a different molecular mechanism and played a different role from that of simple ASEs.

## Methods

We used 5,176 orthologous gene pairs of human and mouse from the EBI database [33] and extracted reciprocal best-hit coding exon pairs using the BLAST package (version 2.2.11 from NCBI website). The human and mouse files used to annotate exon types (including the ASE types) were download from ASD (AltSplice Human Release 2 based on Ensembl 27.35a.1 and AltSplice Mouse Release 2 based on Ensembl 27.33c.1 [30,34]). Based on the above information, also see Table 1, we divided the extracted human-mouse exon pairs into three groups: A. ASE conservation unspecified (i.e., simple/complex ASEs vs. all exons, the ASEs of which the ASE/CSE splicing patterns of the orthologous exons are not limited), B. lineage-specific ASE (i.e., simple/complex ASEs vs. CSEs, the ASEs of which the orthologous exons are CSEs) and C. conserved ASE (i.e., simple/complex ASEs vs. all ASEs) groups. Note that "All exons" include CSEs and all ASEs; whereas "All ASEs" include simple ASEs, complex ASEs, and uncertain ASE type. Groups B and C are subsets of Group A.

## Abbreviations

AS – Alternative Splicing

ASE – Alternatively Spliced Exon

CSE – Constitutively Spliced Exon

FRFP – Frequency of Reading Frame Preservation

## Competing interests

The author(s) declares that there are no competing interests.

## Authors' contributions

TJC conceived the study. FCC analyzed the data. TJC and FCC wrote the draft. Both authors read and approved the final manuscript.

## Appendix

The sequences and exon types of Groups A, B, and C human-mouse orthologous exons analyzed in this study are available at our web site [35].

## References

[B1] Bracco L, Kearsey J (2003). The relevance of alternative RNA splicing to pharmacogenomics. Trends Biotechnol.

[B2] Brett D, Pospisil H, Valcarcel J, Reich J, Bork P (2002). Alternative splicing and genome complexity. Nat Genet.

[B3] Stetefeld J, Ruegg MA (2005). Structural and functional diversity generated by alternative mRNA splicing. Trends Biochem Sci.

[B4] Lipscombe D (2005). Neuronal proteins custom designed by alternative splicing. Curr Opin Neurobiol.

[B5] Lareau LF, Green RE, Bhatnagar RS, Brenner SE (2004). The evolving roles of alternative splicing. Curr Opin Struct Biol.

[B6] Modrek B, Lee C (2002). A genomic view of alternative splicing. Nat Genet.

[B7] Novembre FJ, Saucier M, Anderson DC, Klumpp SA, O'Neil SP, Brown CR, Hart CE, Guenthner PC, Swenson RB, McClure HM (1997). Development of AIDS in a chimpanzee infected with human immunodeficiency virus type 1. J Virol.

[B8] Mironov AA, Fickett JW, Gelfand MS (1999). Frequent alternative splicing of human genes. Genome Res.

[B9] Kan Z, Rouchka EC, Gish WR, States DJ (2001). Gene structure prediction and alternative splicing analysis using genomically aligned ESTs. Genome Res.

[B10] Modrek B, Resch A, Grasso C, Lee C (2001). Genome-wide detection of alternative splicing in expressed sequences of human genes. Nucleic Acids Res.

[B11] Kan Z, States D, Gish W (2002). Selecting for functional alternative splices in ESTs. Genome Res.

[B12] Kampa D, Cheng J, Kapranov P, Yamanaka M, Brubaker S, Cawley S, Drenkow J, Piccolboni A, Bekiranov S, Helt G, Tammana H, Gingeras TR (2004). Novel RNAs identified from an in-depth analysis of the transcriptome of human chromosomes 21 and 22. Genome Res.

[B13] Maniatis T, Tasic B (2002). Alternative pre-mRNA splicing and proteome expansion in metazoans. Nature.

[B14] Black DL, Grabowski PJ (2003). Alternative pre-mRNA splicing and neuronal function. Prog Mol Subcell Biol.

[B15] Lopez AJ (1998). Alternative splicing of pre-mRNA: developmental consequences and mechanisms of regulation. Annu Rev Genet.

[B16] Chen FC, Wang SS, Chen CJ, Li WH, Chuang TJ (2006). Alternatively and constitutively spliced exons are subject to different evolutionary forces. Mol Biol Evol.

[B17] Chen FC, Chuang TJ (2006). The effects of multiple features of alternatively spliced exons on the Ka/Ks ratio test. BMC Bioinformatics.

[B18] Xing Y, Lee C (2005). Evidence of functional selection pressure for alternative splicing events that accelerate evolution of protein subsequences. Proc Natl Acad Sci U S A.

[B19] Modrek B, Lee CJ (2003). Alternative splicing in the human, mouse and rat genomes is associated with an increased frequency of exon creation and/or loss. Nat Genet.

[B20] Cusack BP, Wolfe KH (2005). Changes in alternative splicing of human and mouse genes are accompanied by faster evolution of constitutive exons. Mol Biol Evol.

[B21] Xing Y, Lee C (2005). Assessing the application of Ka/Ks ratio test to alternatively spliced exons. Bioinformatics.

[B22] Resch A, Xing Y, Alekseyenko A, Modrek B, Lee C (2004). Evidence for a subpopulation of conserved alternative splicing events under selection pressure for protein reading frame preservation. Nucleic Acids Res.

[B23] Sorek R, Ast G (2003). Intronic sequences flanking alternatively spliced exons are conserved between human and mouse. Genome Res.

[B24] Hurst LD, Pal C (2001). Evidence for purifying selection acting on silent sites in BRCA1. Trends Genet.

[B25] Filip LC, Mundy NI (2004). Rapid evolution by positive Darwinian selection in the extracellular domain of the abundant lymphocyte protein CD45 in primates. Mol Biol Evol.

[B26] Iida K, Akashi H (2000). A test of translational selection at 'silent' sites in the human genome: base composition comparisons in alternatively spliced genes. Gene.

[B27] Thanaraj TA, Clark F, Muilu J (2003). Conservation of human alternative splice events in mouse. Nucleic Acids Res.

[B28] Philipps DL, Park JW, Graveley BR (2004). A computational and experimental approach toward a priori identification of alternatively spliced exons. Rna.

[B29] Sorek R, Shemesh R, Cohen Y, Basechess O, Ast G, Shamir R (2004). A non-EST-based method for exon-skipping prediction. Genome Res.

[B30] Stamm S, Riethoven JJ, Le Texier V, Gopalakrishnan C, Kumanduri V, Tang Y, Barbosa-Morais NL, Thanaraj TA (2006). ASD: a bioinformatics resource on alternative splicing. Nucleic Acids Res.

[B31] Chen FC, Chaw SM, Tzeng YH, Wang SS, Chuang TJ (2007). Opposite evolutionary effects between different alternative splicing patterns. Mol Biol Evol.

[B32] de Souza SJ, Long M, Klein RJ, Roy S, Lin S, Gilbert W (1998). Toward a resolution of the introns early/late debate: only phase zero introns are correlated with the structure of ancient proteins. Proc Natl Acad Sci U S A.

[B33] The EBI database. http://www.ebi.ac.uk/.

[B34] The ASD database. http://www.ebi.ac.uk/asd/.

[B35] The sequences of exons analyzed in this study. http://www.sinica.edu.tw/~trees/Simple_Complex/Reading_frame.htm.

